# Global and Local Features of Semantic Networks: Evidence from the Hebrew Mental Lexicon

**DOI:** 10.1371/journal.pone.0023912

**Published:** 2011-08-24

**Authors:** Yoed N. Kenett, Dror Y. Kenett, Eshel Ben-Jacob, Miriam Faust

**Affiliations:** 1 Gonda Brain Research Center, Bar-Ilan University, Ramat-Gan, Israel; 2 School of Physics and Astronomy, The Reymond and Beverly Sackler Faculty of Exact Sciences, Tel-Aviv University, Tel-Aviv, Israel; 3 Department of Psychology, Bar-Ilan University, Ramat-Gan, Israel; University of Maribor, Slovenia

## Abstract

**Background:**

Semantic memory has generated much research. As such, the majority of investigations have focused on the English language, and much less on other languages, such as Hebrew. Furthermore, little research has been done on search processes within the semantic network, even though they are abundant within cognitive semantic phenomena.

**Methodology/Principal Findings:**

We examine a unique dataset of free association norms to a set of target words and make use of correlation and network theory methodologies to investigate the global and local features of the Hebrew lexicon. The global features of the lexicon are investigated through the use of association correlations – correlations between target words, based on their association responses similarity; the local features of the lexicon are investigated through the use of association dependencies – the influence words have in the network on other words.

**Conclusions/Significance:**

Our investigation uncovered Small-World Network features of the Hebrew lexicon, specifically a high clustering coefficient and a scale-free distribution, and provides means to examine how words group together into semantically related ‘free categories’. Our novel approach enables us to identify how words facilitate or inhibit the spread of activation within the network, and how these words influence each other. We discuss how these properties relate to classical research on spreading activation and suggest that these properties influence cognitive semantic search processes. A semantic search task, the Remote Association Test is discussed in light of our findings.

## Introduction

Search processes, both conscious and unconscious, are abundant within the cognitive system, across all domains. To note just a few examples – whenever we need to apply various semantic memory tasks, we constantly invoke search processes within the mental lexicon [1]; whenever we try to retrieve a name of someone we know which is on “The tip of our tongue”, we invoke a search within the phonological network [2]; and finally, whenever we are confronted with a problem, we invoke a search process throughout the problem space [3]. All these, and other cognitive search processes, share the underlying assumption that knowledge is organized as a network, where some concepts are closer to each other, while others are farther apart, an assumption that is dominant within semantic memory research. The present study applies correlation and network methodologies to examine a unique dataset of association norms in Hebrew. Further than providing for the first time a quantitative analysis of Hebrew semantics, the analysis presented here revealed global and local network properties which influence semantic search processes.

The classical models of semantic memory, developed in the 1970's [1], [4], have been mainly investigated by gathering association norms. While the empirical collection of association norms has long been established in the clinical sense (for a review see [5]) from the 1970's onwards, the scientific interest in association norms shifted to a dogma in which associations are viewed as a means to explore the structure of the mental lexicon. As this dogma evolved, different frameworks of the mental lexicon were developed, such as the prototype framework based on Rosch's research (i.e. [6]) and the spreading activation framework offered by Collins and Loftus [7].

The spreading activation model for semantic memory presented by Collins and Loftus [7] is a revision of the theory presented by Collins and Quillian [8]. This framework conveys semantic memory as a network in which concepts are represented as nodes and the relationships between these concepts are represented by links, or edges, in the network [9]. Furthermore, they suggest that concepts in semantic memory are organized according to semantic similarity – the more properties two concepts have in common, the more closely related they are in the network.

As a result of challenges to the Collins and Quillian model [8], the revised framework presents a “spreading activation” process: once a specific node (concept) is activated within the network, activation “spreads” to all other nodes which are connected to it. They further suggest that links between concepts can vary in their strength – the speed with which activation spreads from one node to another, activation that decays over time and distance [10]. The relationship between two nodes can thus be described as a function of the path length between them, which represents the associative strength between the two concepts [11], [12]. Thus, the higher the association strength, the shorter the path between these two nodes in the network [11]. Lorch [13] further studied the spreading activation framework and examined how the strength of an association determines the speed in which that association is retrieved [13]. In a series of experiments, Lorch [13] showed that while the strength of an association determines the activation level, it does not determine the duration of the activation. Thus, he concludes, association strength and duration of activation are independent of each other.

Since the introduction of these two classic frameworks for semantic memory in the 1970's, and the extensive research based on them, other computational models of semantic memory have been suggested for semantic memory. A few examples of such computational models are the Latent Semantic Analysis (LSA) and the Hyperspace Analogue to Language (HAL) models, both extract semantic relatedness through the analysis of co-occurances of words within corpora of texts (for an extensive review, see [4]). These models tackle the issue of semantic memory from the analysis of text corpora whereas the prototype model [6] and the spreading activation model [7] tackle this issue from the gathering of data norms, and hence provide different perspectives on semantic memory. Nevertheless, to date there seems to be no unifying model for semantic memory.

In recent years, the Small World Network (SWN) has gained a lot of attention with regard to its description of complex networks. This model [14], [15] refers to networks which are made up of many sub-clusters and relatively short path lengths between these sub-clusters, and has been found to successfully describe a wide range of sociological, technological and biological networks [16]. Two main characteristics of small world networks are the networks clustering coefficient and its average shortest path length. The clustering coefficient refers to the probability that two neighbors (a neighbor is a node *j* that is connected through an edge to node *i*) of a randomly chosen node will themselves be neighbors. The average shortest path length refers to the average shortest amount of steps (nodes being traversed) needed to be taken between any two pair of random nodes. A small world network is characterized by having a large clustering coefficient and a short average shortest path length [16].

The third main characteristic of small-world networks is its degree distribution [P(k)] – the distribution of amount of edges (k) per node in the network. This characteristic is significant due to the fact that complex systems do not abide to the Gaussian (normal) distribution, and rather present scaling law distributions (such as exponential, or power-law) [17], [18]. In fact, the shape of a networks distribution provides a unique and characteristic signature for different kinds of network structure and processes of network growth [19]. Even though scaling laws are abundant in cognitive phenomena, only quite recently has attention been focused on this issue [20]. While small-world structures are essentially defined by the combination of high values of clustering coefficient together with low values of average shortest path length, scale-free structures are characterized by non-Gaussian degree distributions, with fat tails. As such, not all small-world networks are scale-free [20].

In the past few years, the application of the SWN model within neuroscience research has been growing rapidly [21], slowly assimilating into cognitive research. One such cognitive domain that has embraced this analytic perspective is the language domain, and in particular the study of the semantic mental lexicon. This SWN research effort in the realm of semantics is based on the analysis of free association norms [9], [22]. For example, Steyvers and Tenenbaum [19] explored the SWN properties of free associations and other conventional semantic datasets. Recently, a similar analysis was done on Dutch [23], Spanish and German free association norms [9]. Finally, the SWN nature of phonological networks in several languages has recently been investigated [24].

Studying the semantic lexicon with the use of complex network methodology, based on association networks, poses great merit. This is due to the general agreement, from a psychological point of view, that associations are one of the organizing principles of semantic memory [9]. Analyzing the mental lexicon through this perspective may thus contribute to the understanding of memory search processes by exploring the general principles governing the structure of the mental lexicon [19], principles that classic lexicon structure theory models (such as [7]) do not account for. In fact, the research done so far has consistently exhibited the SWN of semantics, and has led to the claim that this SWN organization of the semantic lexicon satisfies cognitive constraints of information retrieval [9]. In this sense, the high clustering coupled with the low average shortest path length in the network allows for fast search and retrieval of information [9]. With the advancement of language research in these directions, efforts have shifted from a general description of language network characteristics to the study of various cognitive phenomena of language [9]. A few examples of such research are developmental processes of semantic acquisition and network growth [19], [25], semantic similarity [26] verbal fluency [27], semantic search [28], [29] and insight [30], [31]. In fact, a small but growing amount of research focuses specifically on search processes within the network [27], [28], [30], which are constrained by the networks topology.

The above mentioned SWN characteristics of the semantic lexicon have previously been investigated in English, Dutch, German and Spanish [9], [19], [23], languages that originate from the same Proto-German family. Thus, analyzing the SWN properties of a non Proto-German language could help generalize these findings. In fact, there is a long standing debate within the cognitive field on the relationship between language and thought, and whether language effects thought, a debate which is far from being decided [32], [33]. Hebrew is a Semitic, very ancient language, that is greatly different (in syntactical and morphological sense) from the Proto-German languages, and in many senses is still true to its biblical form. As such, studying the architecture of the Hebrew semantic lexicon can significantly contribute to research of the mental lexicon and for the first time provide a solid ground for studying semantic processes in Hebrew.

Furthermore, in the present research we employ novel network methodologies to explore global and local features of semantic networks which influence search processes within the semantic network. This was achieved by analyzing a unique dataset of free associations in Hebrew, examining for the first time the characteristics of the Hebrew semantic lexicon. We begin by examining its global network features and by charting the networks' topology. Next we investigate the local features of the network, a process which allows us to observe causal relations between the nodes of the network. We conclude our research by proposing that the global and local characteristics of the network entail cognitive semantic search processes and illustrate our proposal with the Remote Association Test [34], a task which measures semantic creative ability.

## Materials and Methods

### Data

The data analyzed in this study consists of free association norms in Hebrew gathered by [5]. In their study, 60 subjects were presented with target words and had one minute to generate as many association responses as they could to each presented target word. The method used to collect this dataset, therefore, differs from previous word association datasets - in these other datasets, subjects were requested to generate either only one [35] or three associations [23] to a target word. For example, the dataset collected by [35] contains association norms gathered since the 1970's and amounts to nearly three quarters of a million association responses to five thousand target words, and is the largest dataset of free association norms in English. In this dataset, subjects are presented with a target word and are requested to write down the first word that comes to mind which is meaningfuly or strongly related to that target word. As such, subjects report only associations which are strongly connected to the target words and omit associations which are weaker in their associative connection to that target word. However, the method used to collect the dataset analyzed in this research gathers strong as well as weaker associations to target words. This method is superior to previous methods in collecting association norms, as it exposes a greater part of the mental lexicon, and helps to statistically strengthen significant associations to target words within the network. Furthermore, this method conveys a superior way to examine semantic similarity, as defined by Collins and Loftus [7] – the more different properties (association responses) generated to a target word, the more it is possible to relate that target word to different target words in the sample network.

**Figure 1:**
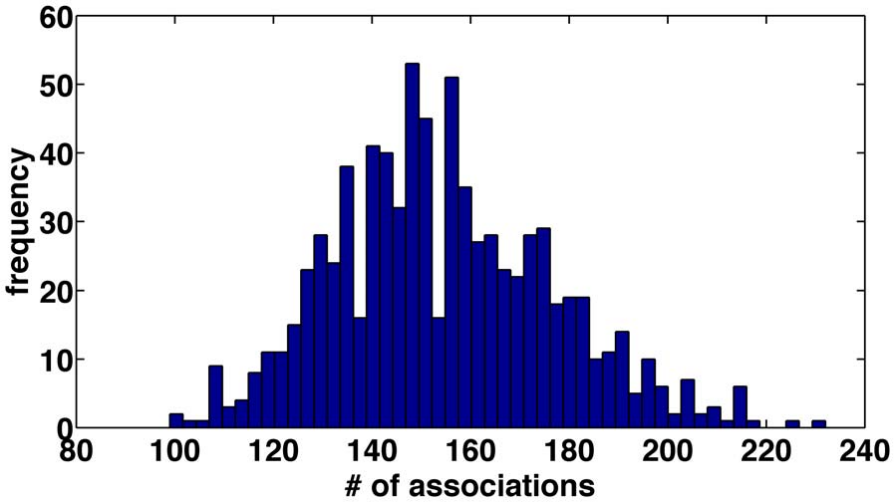
Association Histogram. Histogram of the number of association responses to target words.

In total, the subjects were presented with 800 different target words, in four separate sessions (200 target words in each session; see [5] for a full description). The words equally represented all letters of the Hebrew alphabet, and the number of words beginning in a certain letter was proportional to that letter's share in the Hebrew lexicon. In addition, morphological and semantic considerations were taken into account while compiling the set of target words [5]. On average, each target word received 154 different association responses, which were normally distributed (Figure 1).

### Preprocessing

In order to analyze the dataset, we first standardized the data into a matrix, in which every column is a different target word and every row is a different association response to a target word, deriving a 123664×800 matrix. Since many similar association responses were received for different target words and due to various typing errors within the data, we proceeded to a preprocessing phase in order to construct a matrix where each row was a unique singular association response. This preprocessing stage entailed two actions – standardizing association responses (i.e. neighbour→neighbor; 3.5% of all responses) and converting plural into singular (i.e. fruits→fruit; 13.5% of all responses). Next, all standardized association responses were organized into a single matrix (123664 association responses by 800 target words) and identical association responses were merged using the Minitab software (www.minitab.com), in order to condense the matrix in such way that each row is a single unique association response. This resulted in a 25814 (association responses) by 800 (target words) matrix.

### Global level system analysis

#### Association correlation network. The association correlation matrix

First, we computed the association correlation matrix from the association data. The correlations between the target word associations profiles (the associations of the target words given by all subjects), were calculated by Pearson's formula:
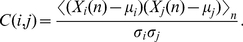
(1)Where 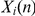 and 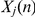 are the associations of word *i* and *j*, and 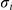 and 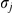 are the STD of the association profiles of target words *i* and *j*, and *n* is the number of possible associations. Note that the target word-target word correlations (or for simplicity the association correlations) for all pairs of words define a symmetric correlation matrix whose 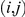 element is the correlation between target words *i* and *j*.

#### Network representation of the association correlations

The association correlation matrix can be studied in terms of an adjacency matrix of a weighted, undirected network. In this view, each target word is a node in the network, and an edge (link) between two nodes (words) is the correlation between these two nodes, with the correlation value being the weight of that link. Thus, the association correlation matrix represents a fully connected weighted network in which the nodes represent the target words, and the links represent the correlations between these words.

#### Informative sub-graphs of the association correlation network

The complete association correlation network for *N* target words contains 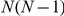 edges. Since most of the edges have small values (weak correlations), the relevant information about the network (e.g. topology, organization), can be obscured. Several methods have been developed to overcome this obstacle by constructing from the complete network a sub-graph that captures the most relevant information embedded in the original network. A widely used method to construct informative sub-graph of a complete network is the Minimum Spanning Tree (MST) [36]–[41]. Another informative sub-graph which retains more information (in comparison to the MST) is the Planar Maximally Filtered Graph (PMFG) [42] which is used here. Both methods are based on hierarchical clustering and the resulting sub-graphs include all the *N* nodes in the network whose edges represent the most relevant association correlations. The MST sub-graph contains 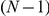 edges with no loops while the PMFG sub-graph contains 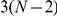 edges.

#### Construction of the PMFG informative sub-graph

To construct the planar maximally filtered graph (PMFG) we first order the 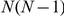 values of the correlation matrix *C* in decreasing rank. We then start from the pairs of nodes, say *i* and *j*, with the highest correlation and draw a link *j*→*I* between them. The process continues according to the rank order where in each iteration a link is added if and only if the resulting graph (network) is still planar, i.e. it can be drawn on the surface of a sphere without link crossing [42]. In the resulted sub-graph, referred to as 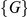, the original values of the correlations are not retained (i.e. all the links have a weight 1). We also note that the sub-graph 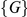 contains (for *N*≫1), 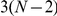 edges – the maximum number of directed edges for planar graph.

### Network parameters

The network parameters calculated were mainly performed with the Brain Connectivity Toolbox for Matlab [43]. The network parameters calculated were the Clustering Coefficient (CC [15]), the average shortest path length (L), the network's diameter (D), and the mean degree number (<k>) [16]. The exponent of the degree distribution (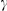) was calculated by the method described in Clauset, Shalizi and Newman [44]. Furthermore, in order to examine the network's clustering coefficient and average shortest path length, a random network was created with the same number of nodes and edges. For this random network, we calculated its clustering coefficient (CCrand) and its average shortest path length (Lrand). Finally, the small-world-ness measure (S; [45]) was calculated to quantitatively and statistically examine the small-world nature of the network. This measure examines the trade-off between the networks clustering coefficient and its average shortest path length and is the first quantitative measure established for examining how much truly a network is “small-worlded”, in the sense that any S>1 entails a SWN.

#### Network Topology

Constructing the association correlation network enables studying its topological properties. First, we made use of Newman's modularity measure [46] to investigate whether the network is made-up of cliques of words, by calculating its modularity index (Q) and its clique index (Ci). In order to verify the cliques found by the modularity algorithm, we classified the target-words a-priori into categories. This classification was based on either a prior categorization research [47], or, when no categorization information existed for a target word, was based on the general category emerging from the top ten association responses generated to the specific target word. This process resulted in 107 different category groups, of various sizes.

#### “Word-centrality” in the semantic network

The semantic network representation allows searching for words that have a significant importance in the semantic lexicon. In network theory, the importance of each node in a given network is quantified using different measures, such as the betweeness measure and eigenvalue centrality [16]. Here we make use of a new concept, the “word-centrality”. We define a quantitative measure of word impact, defined as the difference between the average shortest path of the network after removing word *i* with the average shortest path of the full network,
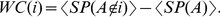
(2)Where *A* is the network adjacency matrix, and 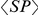 is the average shortest path of the network.

### Local level analysis

#### Dependency Network Analysis

The dependency network approach provides a new analysis of the activity and topology of directed networks. The approach extracts causal topological relations between the network's nodes, and provides an important step towards inference of causal activity relations between the network nodes.

In the case of network activity, the analysis is based on partial correlations, which are increasingly used to investigate complex systems (i.e. [48]). In simple words, the partial (or residual) correlation is a measure of the effect (or contribution) of a given node, say *j*, on the correlations between another pair of nodes, say *i* and *k*. To be more specific, the partial correlations of the 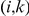 pair, given *j* is the correlations between them after proper subtraction of the correlations between *i* and *j* and between *k* and *j*. Defined this way, the difference between the correlations and the partial correlations provides a measure of the influence of node *j* on the 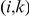 correlation. Therefore, we define the influence of node *j* on node *i*, or the dependency of node *i* on node *j*−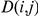, to be the sum of the influence of node *j* on the correlations of node *i* with all other nodes.

#### Partial correlations

The first order partial correlation coefficient is a statistical measure indicating how a third variable affects the correlation between two other variables [49]. The partial correlation between nodes *i* and *k* with respect to a third node *j*−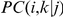 [50] is defined as:
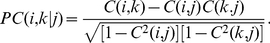
(3)Where 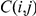, 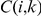 and 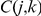 are the node correlations defined above.

#### The correlation influence and correlation dependency

The relative effect of the correlations 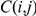 and 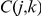 of node *j* on the correlation 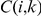 [48] is given by:
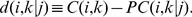
(4)This avoids the trivial case of the node *j* appearing to strongly effect the correlation 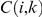, mainly because 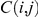, 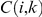 and 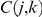 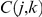 have small values. We note that this quantity can be viewed either as the correlation dependency of 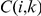 on node *j* (the term used here), or as the correlation influence of node *j* on the correlation 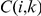.

#### Node activity dependencies

Next, we define the total influence of node *j* on node *i*, or the dependency 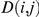 of node *i* on node *j* to be:
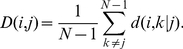
(5)As defined, 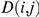 is a measure of the average influence of node *j* on the correlations 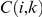, over all nodes *k* not equal to *j*. The node activity dependencies define a dependency matrix *D* whose 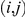 element is the dependency of node *i* on node *j*. It is important to note that while the correlation matrix *C* is a symmetric matrix, the dependency matrix *D* is nonsymmetrical – 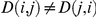 since the influence of node *j* on node *i* is not equal to the influence of node *i* on node *j*.

Note that the association correlation network and the association dependency network target different levels of analysis of the Hebrew lexicon. The association correlation network presents the similarity of target words, according to the association responses provided by the subjects. The association dependency network provides local information on the interaction between words; this network reflects how one word affects the correlations of all other target words. Thus, for example, the nodes dough (‘batzek’) and flour (‘kemach’) have a strong similarity in the association responses given to both words, and thus are connected to each other in the association correlation network (global level). However, the node dough (‘batzek’) does not have a strong influence on the correlations of the node flour (‘kemach’) with all other nodes, and thus these two nodes will not be connected in the association dependency network (local level). The association correlation network provides the global information of the semantic lexicon, whereas the association dependency network provides the local (and potentially causal) information of the semantic lexicon.

## Results

### Association correlation network

We begin by calculating the association correlation matrix. Next, we use the dendrogram hierarchal clustering process [51] to cluster words that have high association correlation. A dendrogram illustrates the arrangements of the clusters produced by the hierarchal clustering process. Dendrograms are usually used in computational biology to illustrate the clustering of genes or samples (i. e. [52]). Here we make use of the Euclidean distance as the distance metric to calculate the dendrogram of the association correlations.

**Figure 2:**
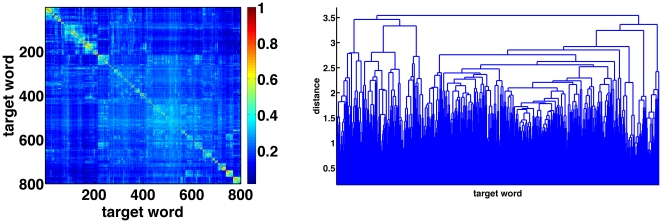
Association correlation matrix. The dendrogram hierarchal clustering method is used to find cliques of words with a strong semantic similarity (left panel), and then to order the normalized association correlation matrix (right panel).

In Figure 2 we present the dendrogram of the association correlation matrix, and the association correlation matrix normalized and ordered according to the dendrogram. We note that using this representation, we observe cliques of words with strong semantic similarity.

Next, we construct the association semantic network from the association correlation matrix, using the PMFG filtering process (see above). We then calculate different SWN properties of the semantic network. The values of the different SWN parameters calculated are summarized in Table 1.

**Table 1:** Summary of results of network analysis: n – number of nodes in the network; L – average shortest path length; D – diameter; CC – clustering coefficient; <k> - mean degree; γ– power-law component; CCrand – Clustering coefficient of random graph; Lrand – average shortest path length of random graph; S – small-world-ness measure; Q – modularity measure; Ci – community index.

Parameter	Value
**N**	800
**L**	10.0349
**D**	25
**CC**	0.6831
**<k>**	5.9425
γ	3.5
**CCrand**	0.0054
**Lrand**	3.9450
**S**	34.3728
**Q**	0.5647
**Ci**	56

These results clearly show the SWN characteristics of the Hebrew association correlation network. The clustering coefficient is much higher than that of the random graph (CC = 0.6831 > CCrand = 0.0054). The small-world-ness measure clearly signifies a SWN (S = 34.37), which was also statistically tested and found significant (see [45] for a description of their significance test method). Unexpectedly, the average shortest path length for the network was higher than that for the random graph (L = 10.0349 > Lrand = 3.94).

**Figure 3:**
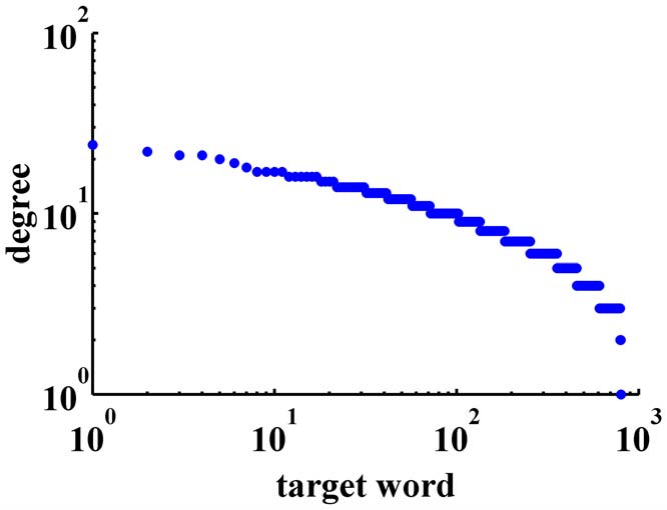
Degree distribution plot. Plot of degree distribution of target words in the correlation network, in a log-log scale.

Examining the degree distribution clearly reveals a non-Gaussian distribution, with a scale-free [19] power law (γ = 3.5). The calculated exponent is within the range of scale-free SWN, as described by Barabási and Albert (17; see also 19]. Figure 3 presents the degree distribution of the nodes in the network.

As can be seen in Table 1, our modularity measure calculation yielded a result of 0.56, suggesting that the data are highly modular and contain many different cliques. The algorithm also divided the data into 56 cliques, significantly lower than our a-priori classification (of 107 different categories). A closer examination of the modularity classification revealed some very large cliques, which contain several sub-cliques mixed together. These differences call for further future research, but might be due to the small sample size of target words out of the entire mental lexicon. Notably, once we plotted the graph of the network (see below), we witnessed several discrepancies in our a-priori classification and the way the target words group together.

### Cliques of the Hebrew association correlation network

**Figure 4:**
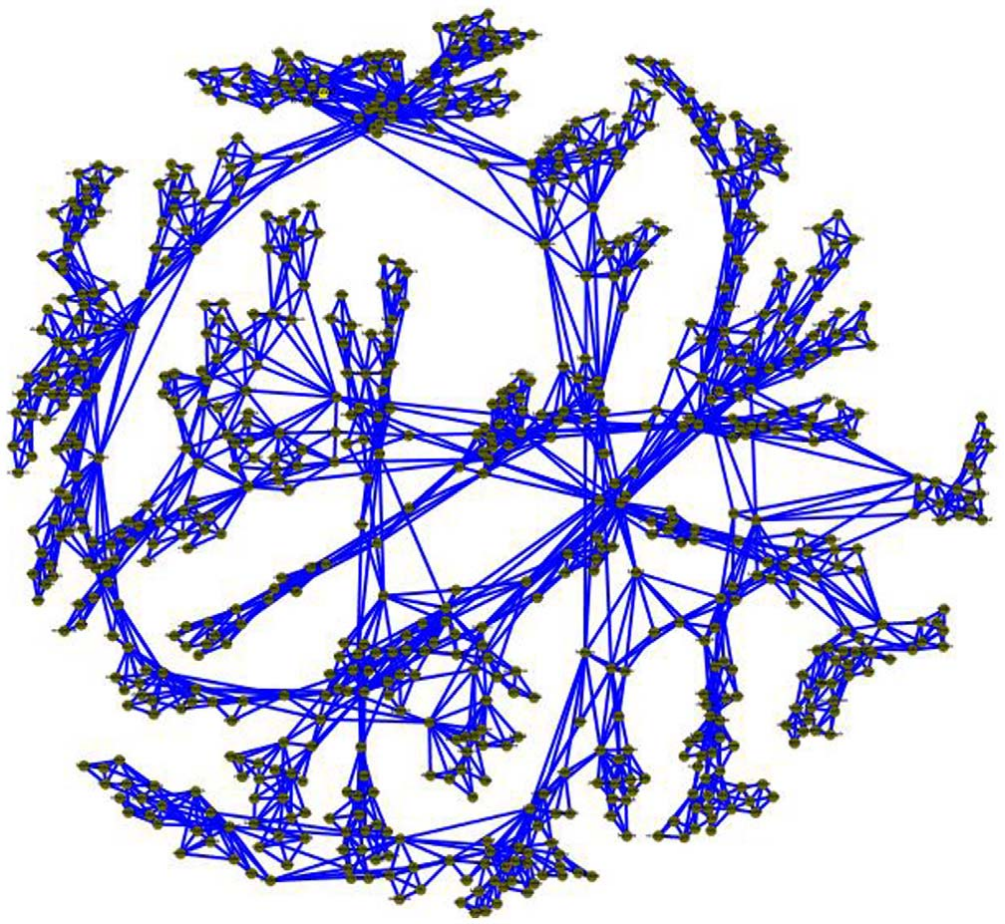
Network 2D visualization. Representation of the entire network of 800 words, as they are grouped together in the planar graph, constructed from the association correlations. Each word is a node in the network (green circle), and a link between two words represents their association correlation (blue line).

**Figure 5:**
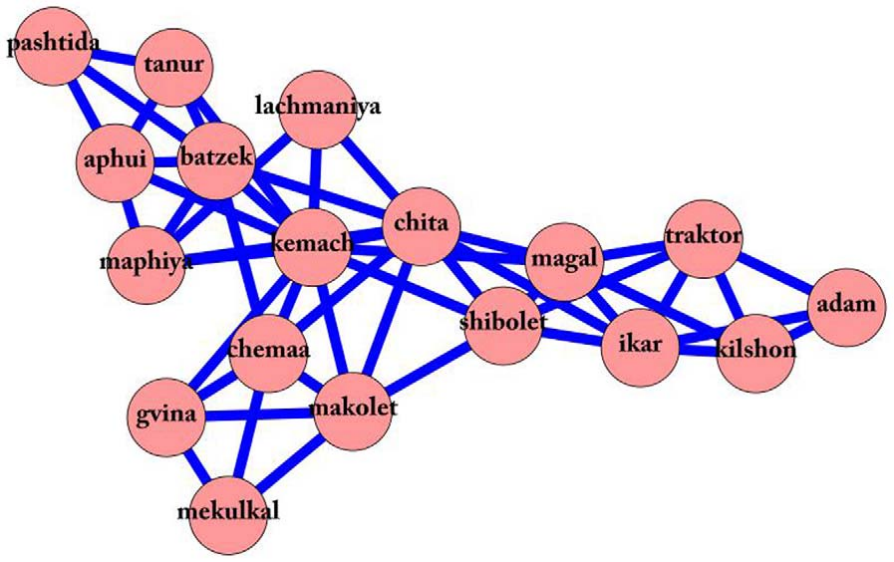
The making bread clique. An example of a clique from the full network, semantically concentrated on the notion of making bread.

To visualize the network we plotted the graph using Cytoscape [53], and in order to present the Hebrew target words as the labels of the nodes, we phonetically transcribed them into English (Figure 4). A close examination of individual cliques shows a strong organization of words by a common semantic category. Following are two such examples:

The clique shown in Figure 5 is dedicated to the meaning surrounding bread making – the word farmer (‘ikar’), who is man (‘adam’), is connected to agricultural tools such as sickle (‘magal’), pitchfork (‘kilshon’) and tractor (‘traktor’), which are needed to plow stocks (‘shibolet’) of wheat (‘chita’). These are connected to flour (‘kemach’), used to make dough (‘batzek’). Next, the dough is baked in the oven (‘tanur’) which results in something that is baked (‘aphui’). This can be a bun (‘lachmaniya’) which can be bought at the bakery (‘maphiya’). Furthermore, in the oven (‘tanur’) some food can be cooked (‘mevushal’), such as a pie (‘pashtida’), for example. Finally, dough can be bought at the store (‘makolet’), along with other groceries such as butter (‘chemaa’) or cheese (‘gvina’), which unfortunately can sometimes get spoiled (‘mekulkal’).

**Figure 6:**
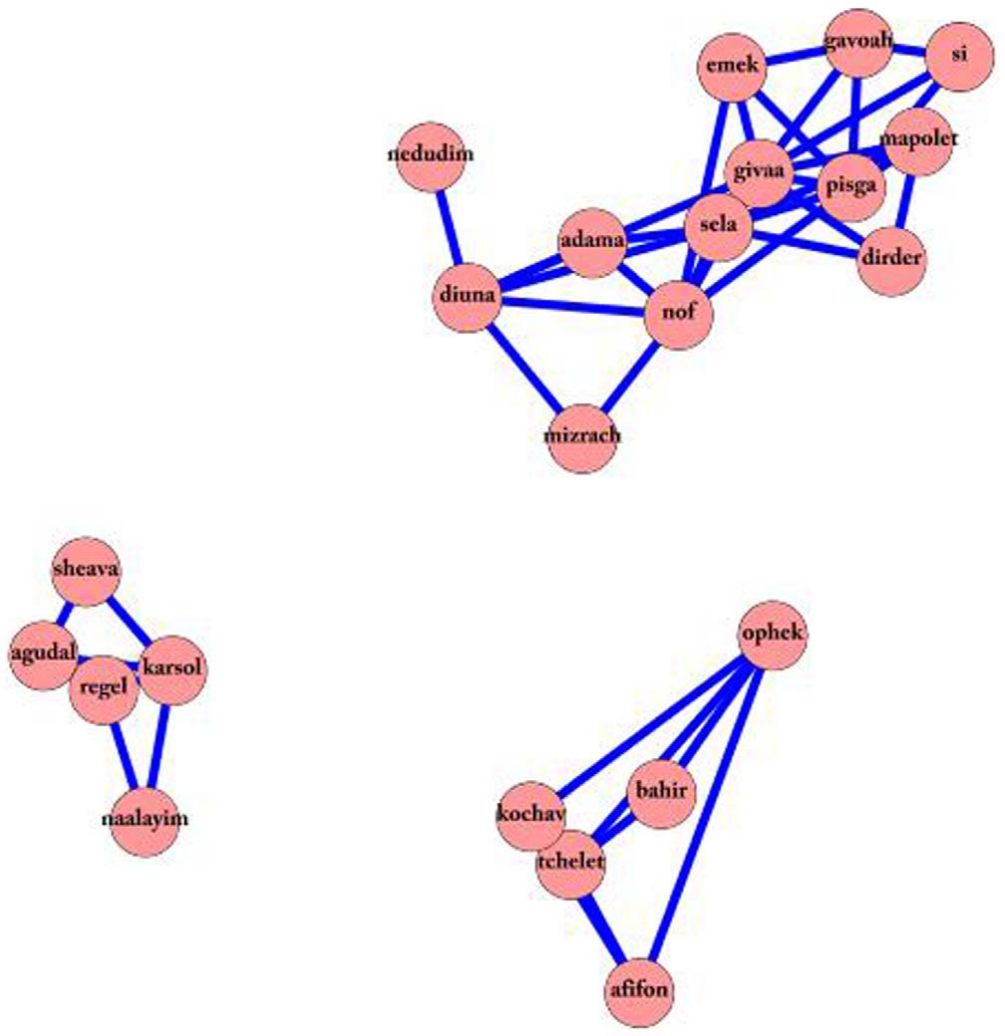
The outdoor cliques. An example of three cliques from the full network, semantically concentrated on foot, sky and hiking. The three cliques are related in their semantic focus, with the left centered on the notion of feet, and the right bottom centered on the notion of the sky, and the top right centered on the notion of hiking.

A second example of cliques within the network is that of three cliques connected to each other in the full network (Figure 6). One clique relates to a person’s foot – regel (‘foot’), thumb (‘agudal’), ankle (‘karsol’), shoes (‘naalayim’) and even wax (‘sheava’); a second clique relates to the sky – bright (‘bahir’), horizon (‘ophek’), light blue (‘tchelet’), star (‘kochav’), and kite (‘afifon’); finally, the third clique relates to hiking – wandering (‘nedudim’), dunes (‘diuna’), earth (‘adama’), scenery (‘nof’), east (mizrach’), rock (‘even’), hill (‘givaa’), valley (‘emek’), summit (‘pisga’), high (‘gavoah’), peak (‘si’), avalanche (‘mapolet’), and rolling down a slope (‘dirder’).

**Figure 7:**
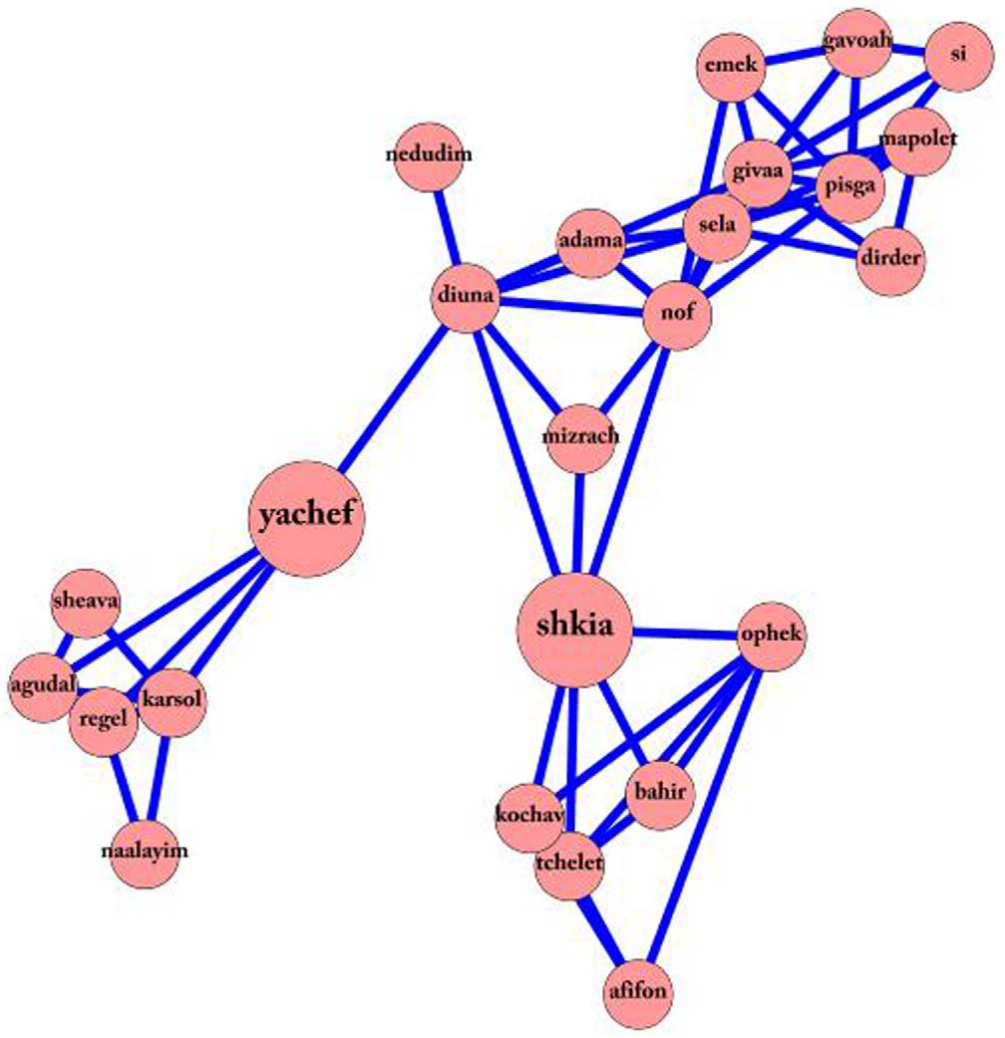
An example of “Gateway nodes”. The cliques presented in Figure 6, concerning the notion of foot, hiking and sky, are connected by two “gateway nodes” – barefoot (‘yachef’) and sunset (shkia’).

These three cliques are connected to each other via two ‘gateway nodes’ (Figure 7) – barefoot (‘yachef’) connecting the foot clique to the hiking clique, and sunset (‘shkia’) connecting the hiking clique to the sky clique. Thus, besides serving as another example of how the target words in the network organize into semantically related cliques, this example also illustrates how the different cliques are connected.

### “Word-centrality” in the Hebrew association correlation network

**Figure 8:**
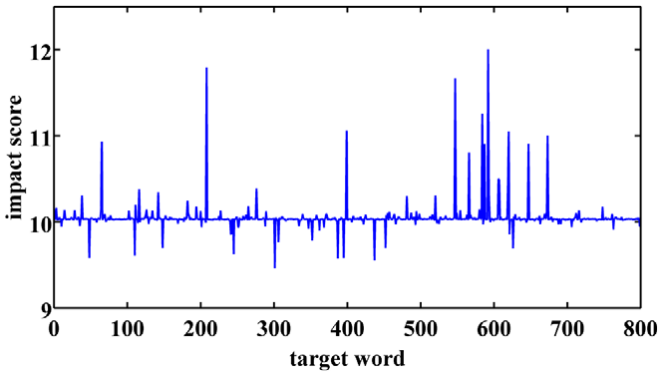
Impact score of the network. The impact of a given word *i* on the semantic network, calculated as the difference between the average shortest path of the full network to that of the network after deletion of the word *i*.

Finally, we investigated the impact of a given word *i* on the semantic network. To this end, we iteratively chose each word and deleted it from the sample, then recalculated the association correlation matrix, the semantic network and finally the average shortest path in the network. We then calculated the impact of each word, as defined above (Figure 8).

As described above, the path length of the network represents the relations between the nodes in the network, and more specifically directly relates to association strength which is a determining factor in the spread of activation [11]–[13]. As such, calculating the impact score of every node measures its general effect on the spread of activation within the network.

A positive impact score signifies that after the deletion of word *i*, the average shortest path length became longer than the average shortest path length of the full network, indicating that this word has a positive effect on the spread of activation within the network. We refer to these words as ‘facilitating hubs’ (FH). In contrast, a negative impact score signifies that after the deletion of word *i*, the average shortest path length became shorter than the average shortest path length of the general network, indicating that this word has a negative effect on the spread of activation within the network. We refer to these words as ‘inhibiting hubs’ (IH). Investigating the impact score of the words in the network, we chose a 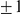 standard deviation (STD) threshold above (below) the mean impact effect (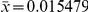), which we deemed as words having significant effect on the network (either facilitative or inhibitive). In our network, we found 22 FH and 15 IH, which are summarized in Table 2.

**Table 2:** Summary of Facilitation Hubs (left table) and Inhibition Hubs (right table).

FH	Impact	IH	impact2
Saad (to nurse)	1.969611	Zricha (sunrise)	−0.57214
Heechil (fed)	1.759909	Mevushal (cooked)	−0.48049
Nedava(donation)	1.628782	Kurkum(turmeric)	−0.45816
Sinor (apron)	1.220627	Itria (noodle)	−0.45045
Kruvit (cauliflower)	1.022254	Kamun (cumin)	−0.44953
Aruga (flowerbed)	1.01617	Bishel (to cook)	−0.42135
Kabtzan (beggar)	0.965875	Histabech (got in trouble)	−0.40847
Asuphi (waif)	0.894084	Poshea (criminal)	−0.34226
Pashtida (pie)	0.868739	Goses (dying)	−0.33363
Salat (salad)	0.866699	Munsham (being ventilated)	−0.33363
Neft (oil)	0.768617	Chol (sand)	−0.26792
Orev (crow)	0.464451	Tipel (treated)	−0.24959
Ataleph (bat)	0.44322	Hanaa (enjoyment)	−0.18504
Atzitz (flowerpot)	0.425491	Hanaka (breast-feeding)	−0.17735
Hityatem (to be orphaned)	0.350795	Arisa (cradle)	−0.1773
Benzin (gasoline)	0.345807		
Givol (stem)	0.306829		
Seara (storm)	0.293871		
Izdarechet (margosa tree)	0.269877		
Miphrasit (sailboat)	0.269191		
Mechonit (car)	0.260143		
Dolar (dollar)	0.212108		

The words are ordered in descending order of their impact strength.

While the importance of FH and IH demands further research, it is interesting to note the FH ‘pashtida’ (pie; impact 0.868739) and the IH ‘mevushal’ (cooked; impact −0.48049). Both connect the clique of bread making to the rest of the network (Fig. 6) but have opposite effects on the spread of activation in the network. This might indicate that activation spreads faster to the clique of bread making through the FH ‘pashtida’, than through the IH ‘mevushal’.

### Association dependency network

**Figure 9:**
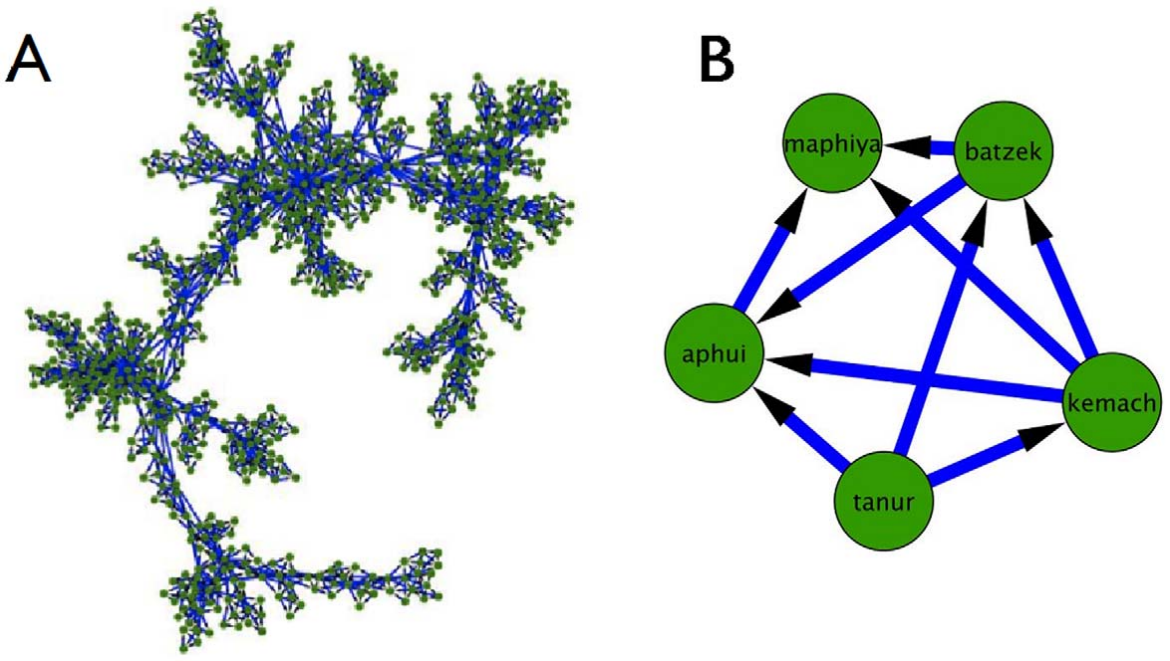
Association dependency network. A 2D visualization of the full association dependency network (left panel), and an example of a dependency clique in the network, showing association dependencies and related to the notion of making bread (right panel).

We constructed the association dependency network from the association correlation matrix, by calculating the partial correlations and then using the PMFG filtering process (see above) to extract the association dependency network, resulting in an 800×800 binary directed network. To inspect the association dependency network topology we plotted the network using Cytoscape [53], presented in Figure 9A.

Exploring the topology of the network reveals a highly modular topology. Calculating the modularity measure [46] returned a value of Q = 0.7334, which confirms the high modularity of the network. Examining these different ‘influence cliques’ reveals that they too (similar to the association cliques) organize around a common semantic theme.

One such influence clique is presented in Figure 9B, and is concerned with the notion of making bread. Unlike the clique presented in Figure 5, this influence clique reveals the influence (or causal) relationship between the different nodes within the clique. As such, in this clique, the node oven (‘tanur’) influences the nodes flour (‘kemach’), dough (‘batzek’) and baked (‘aphui’). The node flour (‘kemach’), in turn, influences the nodes dough (‘batzek’), baked (‘aphui’) and bakery (‘maphiya’). The node dough (‘batzek’) influences the nodes baked (‘aphui’) and bakery (‘maphiya’), and the node baked (‘aphui’) influences the node bakery (‘maphiya’). Note that the node bakery (‘maphiya’) is only influenced by the other nodes in the clique but does not influence any other nodes in the clique.

**Figure 10:**
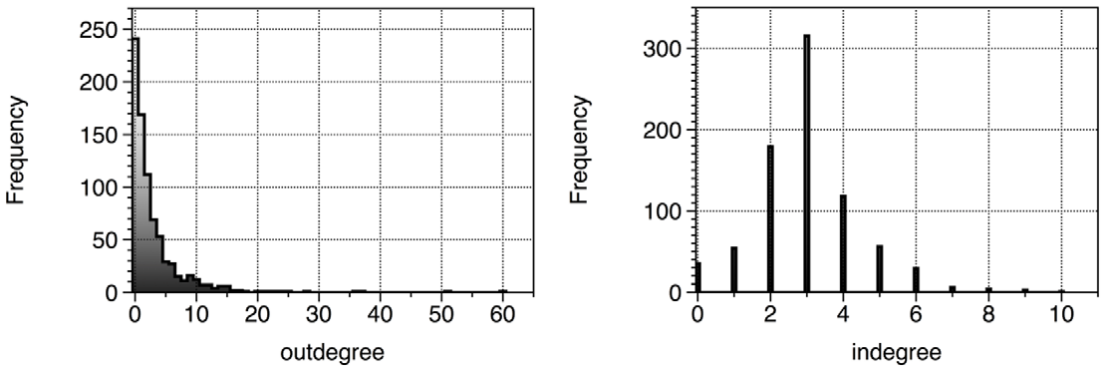
OutDegree and InDegree distributions. OutDegree (left panel) and inDegree distribution (right panel) of node dependency. The outDegree refers to how many nodes are influenced by node *i*, whereas the inDegree refers to how many nodes influence node *i*. The x-label outDegree (or inDegree)refers to to the outDegree (or inDegree) score and the y-label frequency refers to the amount of nodes with that outDegree (or inDegree) score.

On this network we calculated for every node its outDegree, which signifies the influence score of each node (i.e. how many nodes are affected by node *i*); the inDegree, which signifies the affected score of each node (i.e. how many nodes influence node *i*); and the Relative Influence [48], which signifies the relative influence a node *i* has in general within the network, and is defined as
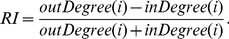
(6)On average, the general outDegree and inDegree of the entire Dependency network are equal (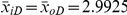), which indicates a stability of influence and effect within the network. However, the outDegree and inDegree distribution are quite different and presented in Figure 10. While the outDegree distribution ranges between 0–60 with a standard deviation of 4.96, the inDegree distribution ranges between 0–10 with a standard deviation of 1.44.

**Figure 11:**
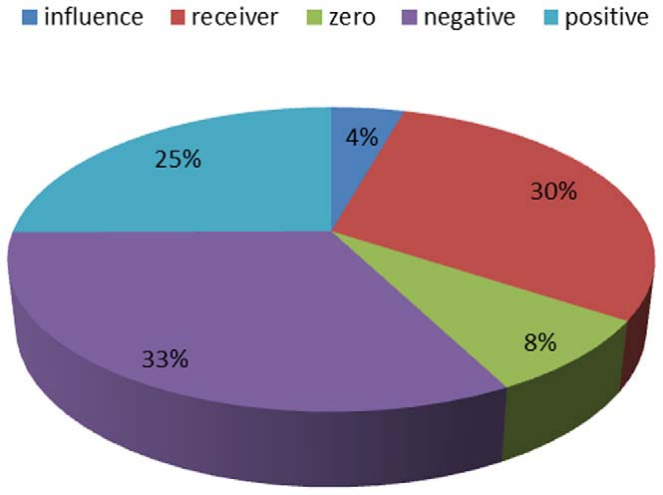
Relative influence score characterization. Percentage of different types of nodes, based on their relative influence score – influence nodes are nodes who have an outDegree > 1 and inDegree = 0; receiver nodes are nodes who have an outDegree = 0 and inDegree > 1; zero nodes are nodes who have an outDegree = inDegree; negative nodes are nodes who have an outDegree < inDegree; and positive nodes are nodes who have an outDegree > inDegree.

In order to examine the differences between the outDegree and inDegree distribution, we analyzed the nodes Relative Influence score, which provides a more objective significance of a node *i* in the network. This analysis resulted in a classification of five different RI node types – nodes that only influence the network and have full influence strength (influence nodes), nodes that only receive influence and have full receiver strength (receiver nodes), nodes that have equal influence-receiver strength (zero nodes), nodes that have a partial influence strength (positive nodes) and nodes that have a partial receiver strength (negative nodes). In our network we found 35 influence nodes (4.375% of the network), 65 zero nodes (8.125% of the network), 201 positive nodes (25.125% of the network), 239 receiver nodes (29.875% of the network) and 260 negative nodes (32.5% of the network). Figure 11 presents the percentage distribution of the different influence nodes.

**Figure 12:**
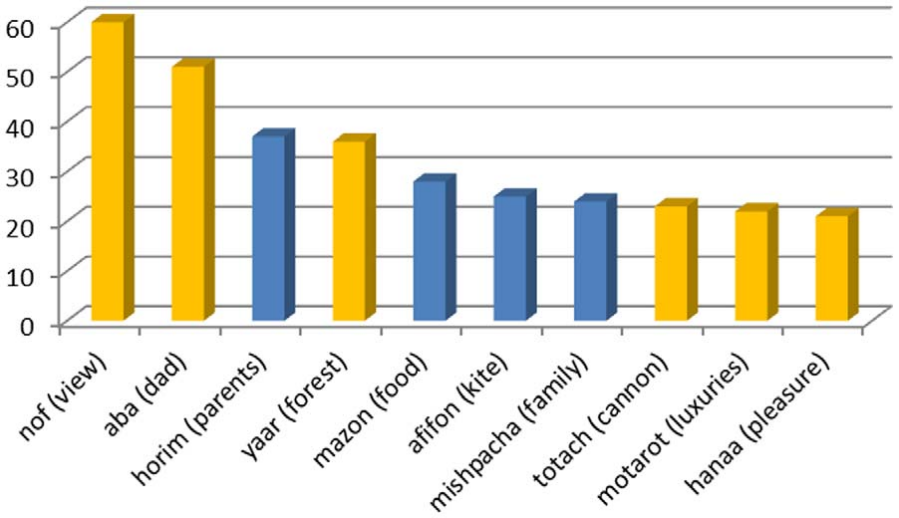
Top 10 strongest nodes based on their outDegree scores. X axis represents the nodes and Y axis represents the outDegree score. Highlighted in orange are nodes which are influence nodes, as described above.

It should be noted that while only 4% of the nodes act as influence nodes in the network, nearly 30% of the nodes act as receiver nodes in the network, and putting the zero nodes aside, there is a 29.5% positive (influence effect) - 52.325% negative (receiver effect) division of the network. This shows that the network influence dynamics is governed by a relatively small number of influence (full or partial) nodes and a larger number of receiver (full or partial) nodes.

table-1-captionWhile the role of the 35 influence nodes is unclear and constitutes only 4% of the entire network, all of these nodes have strong outDegree scores in the network, suggesting that these nodes act as influence hubs in the network. Among the top 10 nodes with the highest outDegree scores (most influential nodes in the network), 60% are such influence nodes. Figure 12 presents the top 10 strongest nodes in the network, according to their outDegree scores, and highlights the influence nodes.

Finally, we compared the results of the association dependency network analysis and that of the association correlation network analysis, by examining the relationship between the Facilitative (Inhibitive) Hubs impact score and their Relative Influence score. While there were only weak correlation coefficients between the RI and the impact score of the Facilitative (Inhibitive) Hubs (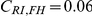 and 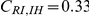), on average the RI of the FH was positive (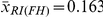) and the RI of the IH was negative (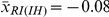).

## Discussion

Here we present a novel approach for studying the global and local features of semantic networks, and apply our approach to examine the Hebrew mental lexicon. The similarities between words based on their free association responses were calculated and used to construct the association correlation matrix. These association correlations were then used to analyze the Hebrew lexicon from a global and local perspective. From the global perspective, this was done by constructing a network representing the Hebrew semantic lexicon and by investigating the characteristics and topology of this network. From the local perspective, this was done by constructing a network which represents the influence effect that different nodes (words) in the network have on each other, and by exploring the characteristics of this influence effect. Furthermore, we investigated the relationship between the global and local levels of the network.

The method used in this research is novel in two ways, the use of free associations and our network analysis technique. The free association dataset analyzed differs from previous free association datasets in the amount of associations generated by subjects per target word. As discussed above, we believe this method may offer a better way to explore the mental lexicon structure, and is in accord with Collins and Loftus [7] notion of semantic similarity. As such, we provide for the first time a quantitative method to explore this notion of semantic similarity. Furthermore, the method we used to extract the most important relations between words in the network (correlations and dependencies) allowed us to deal with the computational complexity of the data, while still retaining the core relationship between the target words.

From the global point of view of the network, we have shown the SWN nature of the Hebrew mental lexicon. This conclusion joins a growing mass of work on the SWN nature of semantics in different languages [9], [19], [23], and supports the notion of the general SWN structure of the mental lexicon. While the average shortest path length of the network was larger than that of the random network (L = 10.034>Lrand = 3.94), which requires further analysis, it must be noted that in the research presented by Steyvers and Tenenbaum [19] and by De-Deyne and Storms [23], all average shortest paths calculated for the networks they analyzed was either equal to or greater than the average shortest path lengths calculated for the random networks.

Furthermore, the construction of the network allows us to identify how the target words organize into sub-cliques, based on semantic categories. Thus, this method revealed how words organize themselves into natural or ‘free’ categories. This is illustrated by the example presented in Figure 7, where cliques concerned with feet, hiking and sky are joined by the words “Barefoot” (‘yachef’) and “Sunset” (‘shkia’). This connection is a probable outcome of the collective past-time of hiking outdoors. Note that the gateway node “Barefoot” is directly connected to the node “dune” (‘diuna’), as people often tend to walk barefoot on dunes, and that the gateway node “sunset” is directly connected to the node “east” (‘mizrach’), which is where the sun rises. We suggest that other research domains studying the organization of the mental lexicon can benefit from these ‘free categories’.

Finally, our calculation of the impact effect of a given word on the general network enables the identification of words that facilitate and inhibit the spread of activation within the network. This impact effect requires further investigation, but can be experimentally used in semantic memory paradigms, in order to investigate the organization of memory and memory retriebal patterns. Furthermore, it can be implemented in the study of individual differences, including clinical populations (e.g. patients suffering from schizophrenia, Asperger or semantic dementia) as a clinical tool.This clinical aspiration is strengthened by a recent study on Autism, which used complex network analysis to investigate neurophysiological differences between autistic and control subjects [55]. This analysis revealed that when compared to a control group pf healthy participants, persons with autism display a smaller Clustering Coefficient, higher average path length and higher modularity index in their functional brain networks.. We expect to find similar differences within their cognitive semantic mental lexicon, and propose that our methods can be used as a tool to map their semantic lexicon and, potentially, lead to treatment protocols that may enhance normal spreading of activation within their semantic lexicon.

From the local system point of view, our analysis of the association dependency network allowed us to explore the local properties of the interaction of nodes within the lexicon. This analysis revealed a balanced influence dynamics of the network, showing that this balanced dynamics is mainly governed by a small amount of strong influence nodes (that only influence other nodes but are not influenced by any other nodes), and by a relatively large amount of ‘receiver’ nodes (nodes that are only influenced by other nodes but do not influence any nodes). Thus, the dependency network exhibits a “scale-free” charactaristic of dependency distribution. This node dependency information can enrich semantic network growth models [19] and may also provide a practical method to investigate language acquisition defecencies in children.

Finally, while the association correlation and dependency networks analyses relate to different and independent levels of the network, we did discover a weak relationship between the two, suggesting that the Facilitative Hubs have a tendency to act as influencing nodes and that the Inhibitive Hubs have a tendency to act more as receiver nodes in the network. These two independent properties of the lexicon (spread of activation and influence strength) are consistent with Lorch's findings, that contradicted the conventional approach that strong associations are activated faster and to a higher level than weak associations [13] and showed the independent effect of association strength and Stimulus Onset Asynchrony (SOA; time interval between prime and probe presentation) on spreading activation. Thus, the global and local network properties reported here present a complementary qunatitative explanation to the different properties of the spreading activation phenomena, as described by Lorch [13].

While previous research examined the SWN of several Proto-German languages and mainly in English [9], [19], [23], this is the first SWN research examining a non Proto-German language – Hebrew. Hebrew is an ancient, Semitic language, which greatly differs in its syntactical and morphological nature from Proto-German languages. As the long standing debate in cognitive research on the relationship between language and thought [32], [33] is far from being over, examining the Hebrew semantic mental lexicon and presenting its SWN nature contributes and strengthens the notion of the universality of semantic network organization, and also offers for the first time a computational analysis of Hebrew semantics which provides a solid ground for similar future research. While the syntactic and morphological properties of Hebrew were not investigated here, our methodology can be used to study these properties. One such possibility is to examine the Hebrew phonological network, and as such expand the work done by Arbesman, Strogatz and Vitevitch [24].

In addition to shedding light on the structure of the Hebrew mental lexicon, these global and local features may explain various semantic cognitive search processes through semantic memory [1]. It is plausible to assume that while commencing the search process through the mental lexicon, the properties of the node which facilitates (or inhibits) the spread of activation and which determines influence strength play a part in the success of the search process. Ergo, the network properties of the semantic mental lexicon discovered here, which were examined on the Hebrew mental lexicon, enable semantic cognitive search processes.

One example of a task entailing a cognitive semantic search is Mednick's Remote Association Test (RAT [34]), focusing on individual differences in verbal creativity.Mednick envisioned the general creative process as “*the forming of associative elements into new combinations which either meet specified requirements or are in some way useful. The more mutually remote the elements of the new combination, the more creative the process or solution*”([34] p. 221). Mednick [34], who defines creativity as the process of combining remote associations, developed the RAT in order to test his theory. In this test, subjects are presented with a triplet of seemingly unrelated words (i.e. *Electric*, *Wheel*, *High*) and are required to find a single fourth word that is related to each of these three words (chair – electric-chair, wheelchair, high-chair; [34]).

We suggest that the network properties of the lexicon described above, combined with the small world theory of insight [30], can explain the search processes undertaken in Mednick's RAT, a notion consistent with his general model of creativity [34]. Once presented with the primed words, the subject must activate a search through the semantic network to find the adjoining target word. If the target word is weakly connected or far away from one or more of the primed words, the search process may not have enough activation strength or ‘get stuck’ within a strongly connected clique of words surrounding one or more of the primed words. Thus, the search cannot be completed. We suggest that the diffusive, high capacity, divergent nature of unconscious thought [54] can facilitate the successful fulfillment of the uncompleted search process, perhaps through the creation of new connections, or through the traversing of different paths within the network [30]. This notion may thus explain the significance of the unconscious phase of creative problem solving known as the incubation phase [31] and is also consistent with Griffiths, Steyvers and Firl [28], who examined the similarities between search processes within the semantic network and the Google search algorithm [29].

In summary, the work presented here adds to a growing mass of work analyzing the SWN nature of the semantic mental lexicon, and is the first such work in the Hebrew language. The method we have used provides a novel way to explore how words organize together and interact with each other within the mental lexicon. We propose that this SWN architecture of the mental lexicon may have significant implications for the understanding of various cognitive semantic search processes, and plan to further explore the results presented here with additional advanced clustering and network methodologies. We will also empirically investigate our results using various semantic paradigms, such as the RAT [34], to explore the nature of semantic search processes, in particular the effect that facilitative and inhibitive hubs have on these semantic paradigms. Finally, as described above, our methods provide practical tools which can be applied in various fields, such as semantic memory, insight problem solving and cognitive processing in clinical populations.

While many questions on the nature of semantic memory and its properties remain open, we propose that bridging together cognitive phenomena such as creativity and the empirically proven Small World nature of the English [19], Dutch [23], Spanish, German [9] and now the Hebrew semantic lexicon, may establish a solid empirical and experimental ground for studying semantic search processes.
